# Research hotspots and trends on NF-κB in cognitive impairment: a bibliometric analysis

**DOI:** 10.3389/fmed.2024.1432455

**Published:** 2024-12-12

**Authors:** Lin Bai, Fangyuan Xu, Peijia Hu, Zhiqiang Shen, Su Xingxing, Qingqing Wang, Hongliang Cheng

**Affiliations:** ^1^The Second Affiliated Hospital of Anhui University of Chinese Medicine, Hefei, China; ^2^Anhui University of Chinese Medicine, Hefei, China

**Keywords:** NF-κB, cognitive impairment, bibliometric analysis, CiteSpace, VOSviewer

## Abstract

**Background:**

Cognitive impairment (CI) endangers the physical and mental health of patients in a significant manner, and it is expected that the number of people with CI in China will rise to 45.33 million by 2050. Therefore, CI has become a popular research topic. Inflammatory damage plays a key role in the pathogenesis of CI, and NF-κB is an important inflammatory signaling pathway. However, no bibliometric analysis regarding the relationship between CI and NF-κB has been reported.

**Methods:**

A bibliometric analysis regarding NF-κB and CI from 1 January 2008 to 12 December 2023 was conducted in the Science Citation Index-Expanded of the Web of Science Core Collection. The frontiers, hotspots, and trends of research regarding the role of NF-κB in CI were identified. VOSviewer and CiteSpace were used to analyze the retrieved articles and identify the author, country, institution, and keywords, as well as co-cited authors, co-cited journals, and co-cited references.

**Results:**

We analyzed 1,468 original articles and reviews. Publications on NF-κB in CI began in 2010 and increased sharply in 2018. Hong Hao was the most represented author, having published 19 articles, and Chinese authors published more studies than those from other countries. China Pharmaceutical University published the most papers; however, the United States has a strong influence and demonstrates international cooperation. The keywords “apolipoprotein e” and “therapeutic target” demonstrated strong citation bursts, and this tendency may persist in the upcoming years. Neuroinflammation demonstrated a strong influence in research regarding NF-κB in CI. Gut microbiota and ketogenic diet also play an important role in NF-κB in CI.

**Conclusion:**

This bibliometric analysis and visualization using VOSviewer and CiteSpace revealed that the role of NF-κB in CI has become a research hotspot. The results of this study indicated that “neuroinflammation,” “microglial,” and “pathway” remain hotspots for future research. However, studies regarding NF-κB in CI have predominantly focussed on basic research; future research should include therapeutic targets, microbiota, and ketogenic diet.

## 1 Introduction

A decline in memory was considered a major manifestation of cognitive impairment (CI) by Willoughby in 1929 (1). Since then, several studies have shown that individuals with age-related memory impairments have longer processing times (2). Modern research indicated that there are various types of CI, including mild cognitive impairment (MCI), functional cognitive impairment (FCI), and dementia. Mild cognitive impairment (MCI) is an intermediate phase between normal aging and dementia (3). FCI refers to patients with subjective persistent cognitive decline, but no objective signs and no association with other organic diseases (4). Dementia is an acquired syndrome in which a person’s cognitive ability is significantly reduced and significantly affects work and life (5). Since 2000, the prevalence of dementia has significantly decreased, which may be due to the increased attention paid by middle-aged and elderly patients to the prevention of cardiovascular and cerebrovascular disease risk factors, which has led to a decrease in vascular dementia (6). However, cognitive impairment remains an important factor affecting the self-care ability, social function, and quality of life of patients (7). Currently, there are approximately 55 million individuals with CI worldwide, and this number is expected to quadruple by 2050 (8).

Studies have shown that inflammatory damage is a key factor in the pathogenesis of CI (9). Microglial activation is a key initiating factor in the central inflammatory response (10). Cellular senescence in neurons and microglia is central to brain aging (11). Microglial activation serves a dual role, as microglia function as macrophages by engulfing damaged cell debris and removing antigenic material (12) while also activating the NF-κB signaling pathway to promote the synthesis and release of pro-inflammatory factors, leading to inflammation (13). Both acute and chronic inflammatory mechanisms regulate cognitive function throughout the life cycle (14). The nuclear factor NF-κB pathway has long been considered a prototypical pro-inflammatory signaling pathway (15). Therefore, the NF-κB pathway may be associated with CI.

Bibliometrics is a discipline that uses mathematics and statistics to analyze the distribution, quantitative relations, and change patterns in literature (16). The visual graph generated by bibliometric software reflects research hotspots and development trends in a certain field (17). Recently, researchers have made remarkable progress in the study of the role of NF-κB in CI, providing a broader perspective on the problems (18). However, several challenges remain to be addressed. In the current study, CiteSpace and VOSviewer software were used to conduct a bibliometric and visual analysis of articles regarding the role of NF-κB in CI that were published from 2008 to 2023.

## 2 Materials and methods

### 2.1 Literature source and data retrieval methods

**Figure 1:**
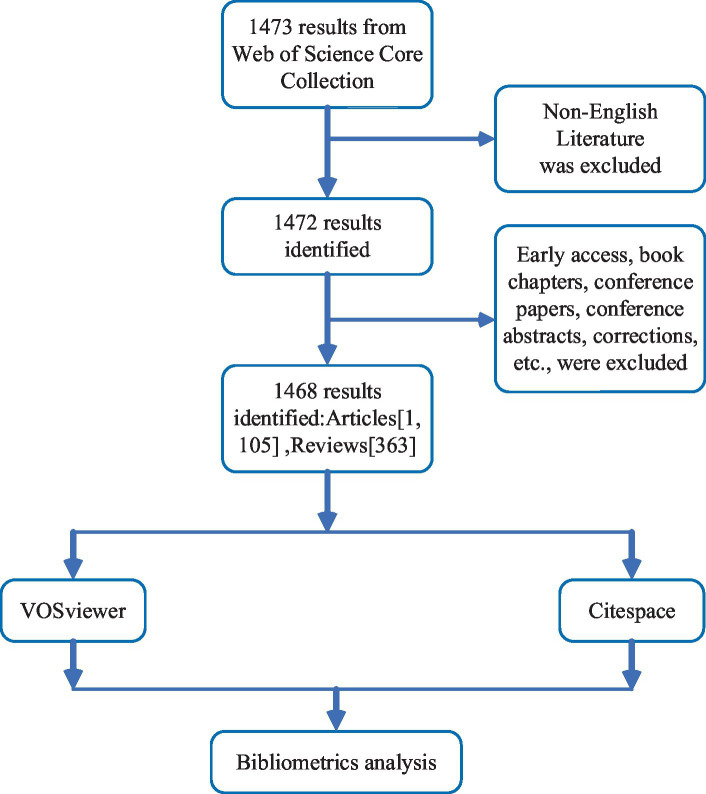
The flow chart for screening the included literature.

A comprehensive retrieval utilizing the core collection of the Web of Science database was conducted on 12 December 2023 to identify studies pertinent to the role of NF-κB in CI. The search formula used was as follows: (TS = (“cognitive impairment” OR “Cognitive Dysfunctions” OR “Cognitive Disorder” OR “Mild Cognitive Impairment” OR “Cognitive Decline” OR “Mental Deterioration” OR “cognitive deficit”)) AND TS = (“NF-κB” OR “NF kB” OR “NF-KB” OR “NF-kappaB” OR “NF kappaB” OR “Nuclear Factor Kappa b” OR “Transcription Factor NF-kB” OR “Transcription Factor NF kB” OR “Nuclear Factor-Kappab” OR “Nuclear Factor Kappa b” OR “nuclear transcription factor-κB” OR “NF-kappa B Complex” OR “NF kappa B Complex” OR “nf kappa b”). All studies included in this analysis were published in English, and invalid documents, such as early access reports (n = 2), book chapters (n = 1), and meeting abstracts (n = 1), were excluded from the analysis. In total, 1,468 papers were retrieved, including articles (n = 1,105) and reviews (n = 363). A flowchart for screening the included studies is shown in Figure 1. The retrieved publications were exported with full records in TXT form in preparation for the bibliometric analysis.

### 2.2 Data analysis

The bibliometric analysis was conducted using CiteSpace (V6.2.R6; manufacturer name and location) and VOSviewer (V1.6.18; manufacturer name and location) software, both based on Java. CiteSpace is used to extract data from a large literature database to help researchers find relevant information to identify trends in a field (19). CiteSpace was used to generate co-occurrence and clustering maps of the references. VOSviewer was used to construct cooperation or co-occurrence networks of authors, countries, institutions, journals, and keywords. VOSviewer has powerful graphical display capabilities and can be adapted for large-scale data (20). Keywords and references with strong citation bursts were displayed to capture emerging tendencies. In this analysis, betweenness centrality served as an indicator for evaluating the significance of nodes in the visualization network. Centrality is a key indicator used to measure the positions of nodes in connected networks. Nodes with high intermediate centrality are often located between large clusters. Nodes with centrality >0.1 are regarded as key points. Productivity is represented by the number of papers (Np), and impact is represented by the number of citations (Nc) (21). The H-index was used to assess the researchers’ study capabilities and expected contributions (22).

## 3 Results

### 3.1 Publication trends

**Figure 2:**
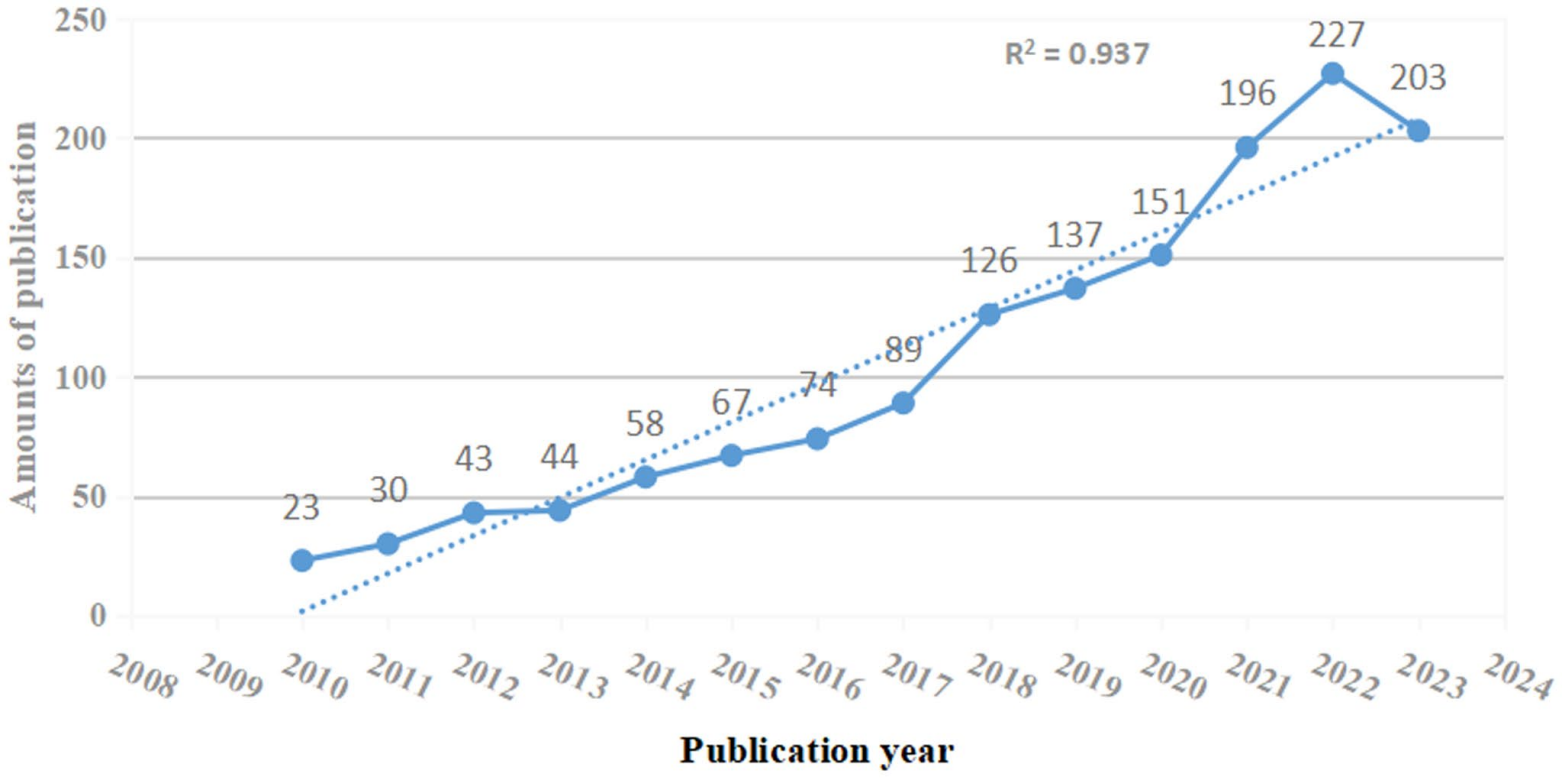
Annual number and trend of publications.

This bibliometric analysis included 1,468 studies regarding the role of NF-κB in CI, including 1,105 articles (75.27%) and 363 reviews (24.83%). As depicted in Figure 2, the number of publications regarding the role of NF-κB in CI increased from 2008 to 2023. However, few studies were published before 2010, indicating that the study of the correlation between CI and NF-κB is novel and growing.

Notably, the number of publications increased rapidly in 2018 and 2021. The peak number of publications was 227 in 2022, and 203 studies were published in 2023. This trend suggests increasing scholarly emphasis on the role of NF-κB in CI, and additional research regarding the role of NF-κB in CI is expected as this field is a research hotspot.

### 3.2 Analysis of authors

A total of 8,209 authors contributed to the 1,468 articles analyzed in this study. According to Price’s Law, core authors studying the role of NF-κB in CI should have published more than four papers (m = 0.749× √ nmax, and nmax = 19). Half of the papers in this analysis were produced by highly productive authors, and 46.93% (*n* = 689) were published by the 130 core authors.

**Figure 3:**
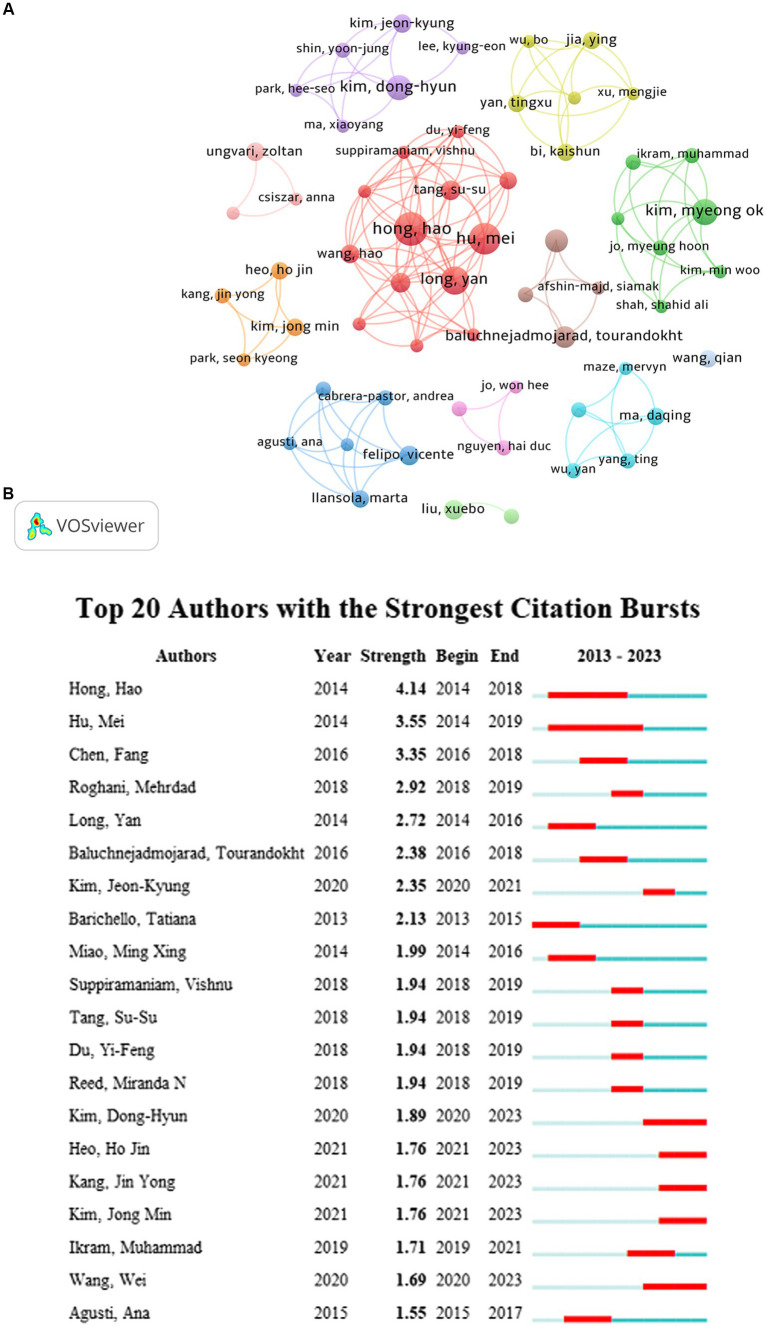
**(A)** The collaboration network map of core authors about NF-κB in CI. **(B)** The 20 authors with the highest number of citation bursts.

Eleven collaborations between the cited authors showed little relationship (Figure 3A). The main studies of the core authors were published between 2016 and 2022, indicating that different groups have the potential for further collaboration. Figure 3B shows the 20 authors with the highest number of citation bursts. Hong Hao began research on cognitive impairment and NF-KB during the earlier period (2014–2018) and had the highest burst strength. Since 2000, an increasing number of new researchers have joined this field of study, and their research is ongoing. These results indicate that research regarding CI and NF-KB is a hotspot with potential for further research.

As shown in Table 1, Hong Hao was the most prolific author among the core authors, publishing 19 articles from 2014 to 2018 and being cited 664 times. Hong Hao reported that some drugs reduce hippocampal Aβ (1–38) and Aβ (1–40) and improve hippocampal plasticity and cognitive dysfunction by inhibiting neuroinflammation and apoptosis mediated by NF-κB signaling pathways (23–25). Studies by Liu Xuebo were cited a mean of approximately 59 times, indicating that these authors were influential. Liu Xuebo’s research demonstrated that reduced memory impairment and amyloid formation can be achieved through NF-κB transcription, regulating the levels of inflammatory mediators and cytokines (26–28).

**Table 1:** The top 10 core authors with the most publications.

Rank	Author	Documents	Citations	Average citations
1	Hao H	19	664	35
2	Mei H	17	627	37
3	Yan L	14	480	34
4	Myeong K	13	551	42
5	Dong-hyun K	12	245	20
6	Mehrdad R	10	371	37
7	Baluchnejadmojarad T	9	355	39
8	Fang chen	8	324	40
9	Vicente F	8	266	33
10	Xuebo L	8	471	59

### 3.3 Analysis of countries and institutions

**Figure 4:**
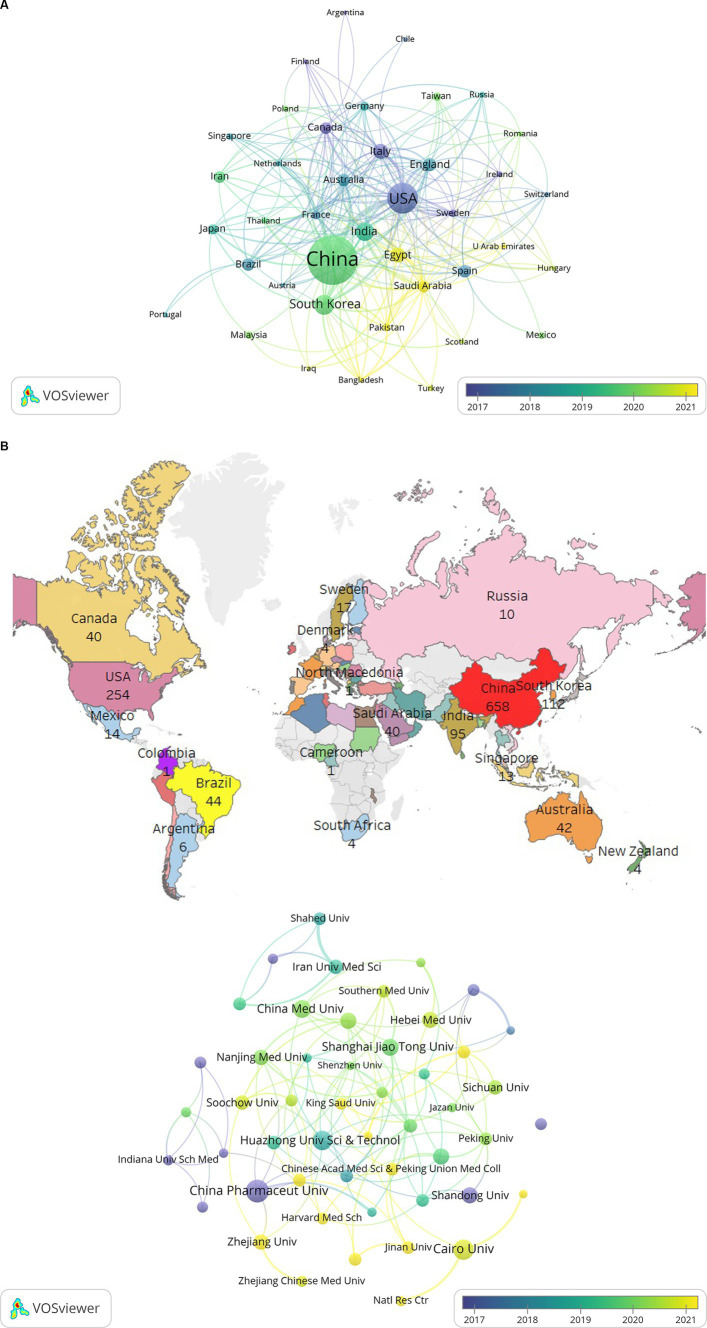
**(A)** The cooperation network map of countries about NF-κB in CI. **(B)** The geographical distribution of the research output about NF-κB in CI. **(C)** The cooperation network map of institutions about NF-κB in CI.

A total of 77 countries published studies regarding the role of NF-κB in CI from 2008 to 2023. The cooperation map of the countries generated using VOSviewer is shown in Figure 4. Figures 4A,B show the main cooperation network map of nations and the geographical distribution of the research output. Table 2 presents the top 10 high-output countries/regions, ranked according to the Np. China produced the most studies (*n* = 658), including 84 basic research papers, 23 clinical research papers, and 86 reviews, indicating that this country has made great contributions to the study of NF-κB in CI. The United States published 254 studies, including 18 basic research papers, 53 clinical research papers, and 96 reviews. South Korea published 112 studies, including 19 basic research papers, 19 clinical research papers, and 18 reviews. Studies from these three countries accounted for 69.8% of the articles included in this analysis. The United States had published the most papers until 2015, though China had the highest Np since 2016, indicating the potential for research from China.

**Table 2:** The top 10 high output countries/regions ranked according to the Np.

Rank	Country	NP	NC	Total link strength	Organization	Np	Nc	Total link strength
1	China	658	16,317	128	China Pharmaceut Univ	34	1,445	10
2	United States	254	14,717	182	Cairo Univ	27	573	11
3	South Korea	112	3,121	52	Huazhong Univ Sci and Technol	24	668	9
4	India	95	3,375	70	China Med Univ	20	514	6
5	Italy	65	3,155	62	Shanghai Jiao Tong Univ	18	1,005	8
6	Egypt	60	1,033	51	Shandong Univ	17	771	4
7	England	50	3,039	61	Hebei Med Univ	17	401	4
8	Brazil	44	2,112	22	Capital Med Univ	17	311	8
9	Australia	42	2065	64	Zhengzhou Univ	16	281	11
10	Spain	42	4,837	21	Zhejiang Univ	15	239	8

China, the USA, and India had the greatest total cooperation, with link strengths of 128, 182, and 70, respectively (Table 2), indicating that these countries maintained close associations with other countries. China was the most prolific country, though its total link strength was not as high as that of the USA. Therefore, China should strengthen its global communication and cooperation for research regarding NF-κB in CI.

Studies from 1,794 institutions were included in this analysis (Table 2). China Pharmaceutical University had the highest output (*n* = 34), followed by Cairo University (*n* = 27), Huazhong University Science and Technology (*n* = 24), and China Medical University (*n* = 20). Iran University of Medicine Science demonstrated the highest total link strength (14), followed by Cairo University, Zhengzhou University, Guangzhou Medical University, and King Abdulaziz University, each with a link strength of 11. These results indicate that although Chinese institutions have the largest number of publications, their link strengths are low. Institutions that published at least nine articles are displayed in Figure 4C. The relative independence of these clusters provides evidence that cooperation among institutions must be strengthened.

### 3.4 Analysis of papers, journals, and co-cited journals

**Figure 5:**
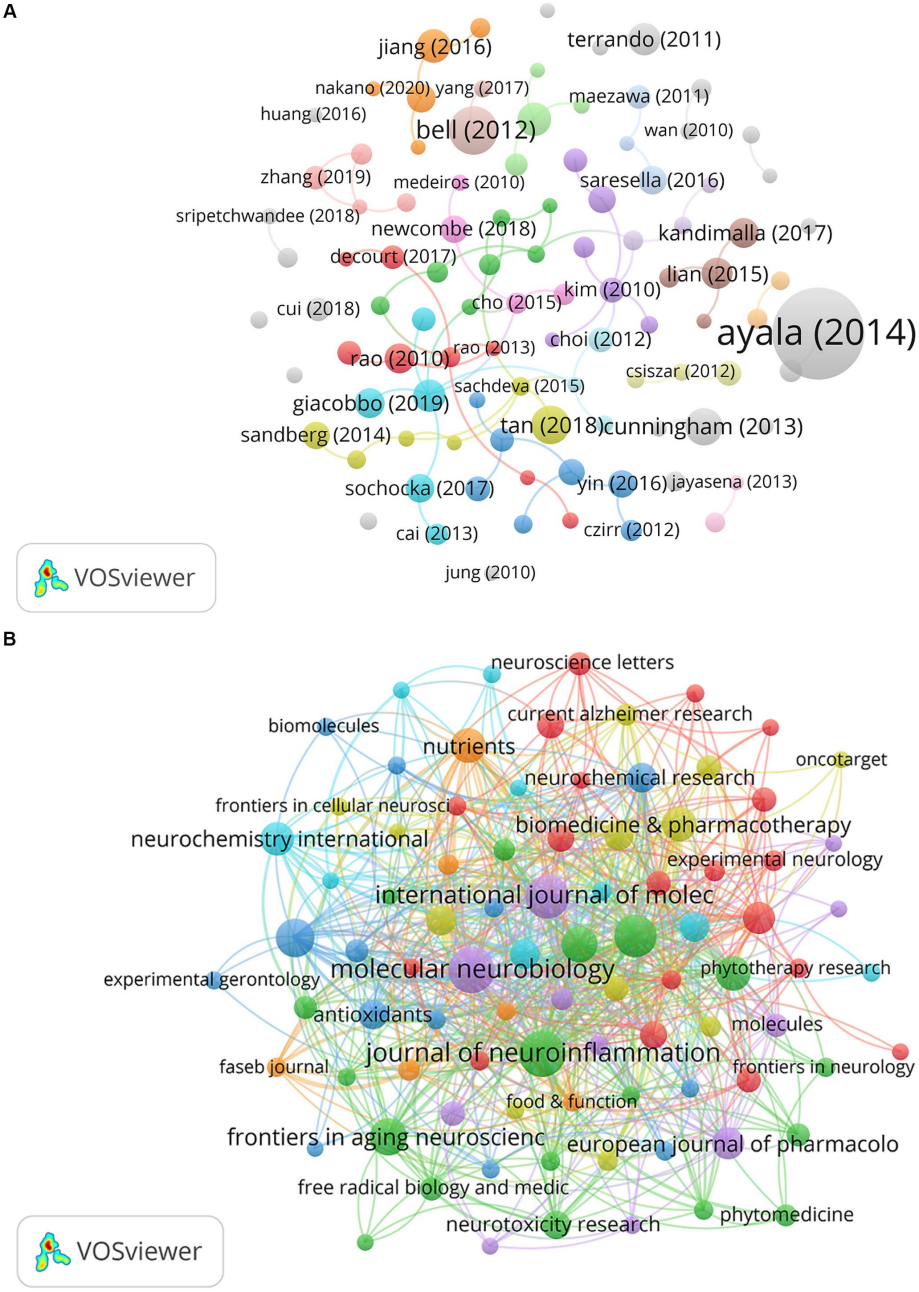
**(A)** The network visualization map of papers about NF-κB in CI. **(B)** The co-occurrence network map of journals about NF-κB in CI.

The co-occurrences of the networks of the studies included in this study are shown in Figure 5A. Due to the large number of papers, the minimum citation number was set to 88. Therefore, 148 of the 1,468 papers were selected for this analysis. A study published by Ayala in 2014 had the highest number of citations (n = 3,114). The journal co-occurrence network is shown in Figure 5B. The minimum number of citations was set to five. Therefore, 88 of the 431 journals were selected for this analysis. *Molecular Neurobiology*, *Journal of Neuroinflammation*, *International Journal of Molecular Sciences*, and *International Immunopharmacology* published numerous papers and exhibited a high influence in this field. The top 10 active journals are listed in Table 3.

**Table 3:** The top 10 active journals ranked according to the Np.

Rank	Journal	Np	Nc	Total link strength	2022 JCR (IF)
1	Molecular Neurobiology	38	2082	75	Q2 (5.1)
2	Journal of Neuroinflammation	37	1956	105	Q1 (9.3)
3	International Journal of Molecular Sciences	34	768	46	Q1 (5.6)
4	International Immunopharmacology	31	660	78	Q2 (5.6)
5	Frontiers In Pharmacology	27	1,234	44	Q1 (3.8)
6	Frontiers in aging Neuroscience	25	754	30	Q2 (4.8)
7	Oxidative Medicine and Cellular Longevity	23	4,251	53	Q2 (5.1)
8	Biomedicine and Pharmacotherapy	22	662	50	Q1 (7.5)
9	Nutrients	22	271	46	Q1 (5.9)
10	Journal of Alzheimer’s Disease	21	727	25	Q2 (3.5)

Among these journals, *Molecular Neurobiology* had the highest Np (n = 38), and *Oxidative Medicine and Cellular Longevity* had the highest Nc (4,251). The *Journal of Neuroinflammation* and *International Journal of Molecular Sciences* also had high Np and Nc values, suggesting that they are highly valuable for research regarding the role of NF-κB in CI. All of the top 10 most active journals belonged to the Q2 (six journals) or Q1 (four journals) partitions, indicating that research regarding the role of NF-κB in CI published in these journals is relatively detailed. However, the impact factors (IFs) of the top 10 most active journals were not high, as 90% of the journals had an IF between 3.5 and 5.9. Therefore, the impact of research regarding the role of NF-κB in CI published in these journals was not sufficient.

**Figure 6:**
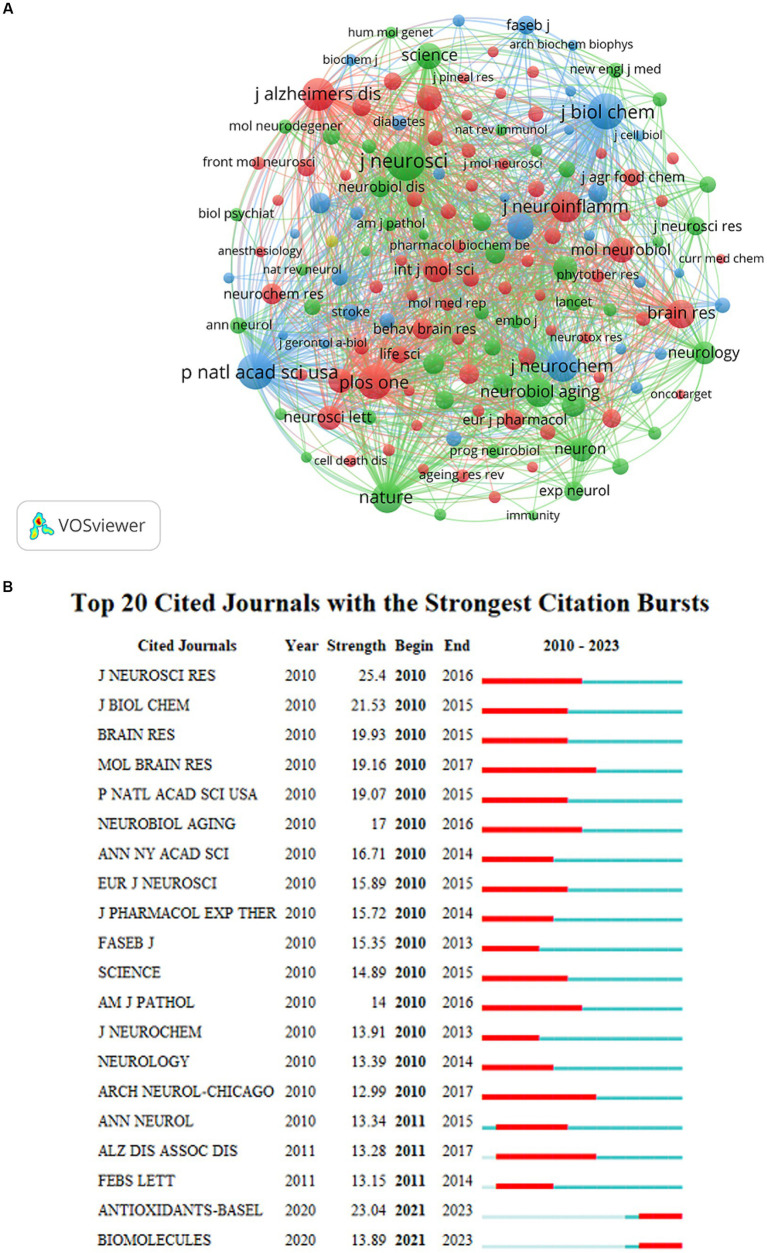
**(A)** The visualization map of co-cited journals about NF-κB in CI. **(B)** The top 20 cited journals with the strongest citation bursts about NF-κB in CI.

Due to the large number of co-cited journals, the minimum number of co-cited journals was set at 200. Therefore, 151 of the 7,552 co-cited journals were included in this analysis (Figure 6A). *J Neurosci*, *J Biol Chem*, and *Nature* were identified as the most frequently cited journals. Figure 6B shows the most representative journals in terms of burst time, duration, and strength. *Antioxidants (Basel)*, *Biomolecules,* and *Brain Sci* published articles regarding the role of NF-κB in CI from 2021 to present. *Antioxidant-Basel* had the second-highest burst strength, with the potential to rank first in burst strength in the near future.

### 3.5 Analysis for co-occurrence and citation burst of keywords

**Figure 7:**
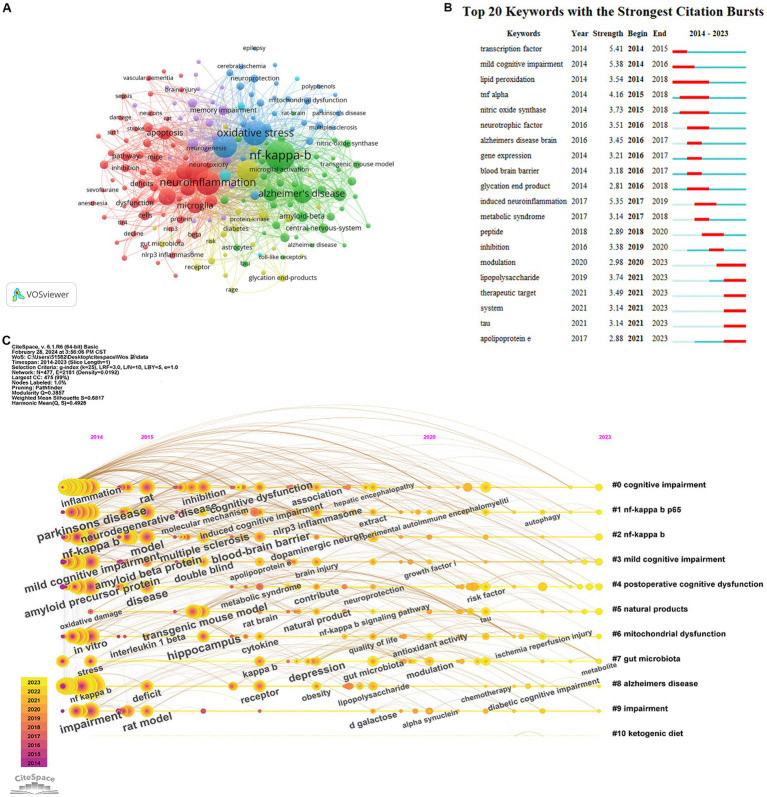
**(A)** The co-occurrence map of keywords about NF-κB in CI. **(B)** The top 20 cited keywords with the strongest citation bursts about NF-κB in CI. **(C)** The research about NF-κB in CI are sorted into chronological order.

VOSviewer was used to display the co-occurrence of keywords. A total of 6,117 keywords were identified, and the minimum number of keyword occurrences was set to 15. Therefore, 186 keywords were included in this analysis. Figure 7A shows that the keywords with the highest frequency were “nf-kappa-b,” “oxidative stress,” “neuroinflammation,” “inflammation,” “Alzheimer’s disease,” “cognitive impairment,” “activation,” “brain,” and “microglia.” Figure 7B shows the most representative keywords in terms of burst time, duration, and strength. “Transcription factors,” “induced neuroinflammation,” and “mild cognitive impairment” are keywords associated with higher burst strength. Keywords “apolipoprotein e” and “therapeutic target” have strong citation bursts, and this tendency may persist in the near future. These results highlight the diversity among the research regarding the role of NF-κB in CI.

Eleven clusters were identified on the clustering timeline (Figure 7C), including “cognitive impairment,” “nf-kappa b p65,” “nf-kappa b p65,” “mild cognitive impairment,” “postoperative cognitive dysfunction,” “natural products,” “mitochondrial dysfunction,” “gut microbiota,” “Alzheimer’s disease,” “impairment,” and “ketogenic diet.” Neuroinflammation demonstrated a high influence in NF-κB in CI. Gut microbiota and ketogenic diet also play an important role in this field of research.

### 3.6 Analysis of co-cited authors

**Figure 8:**
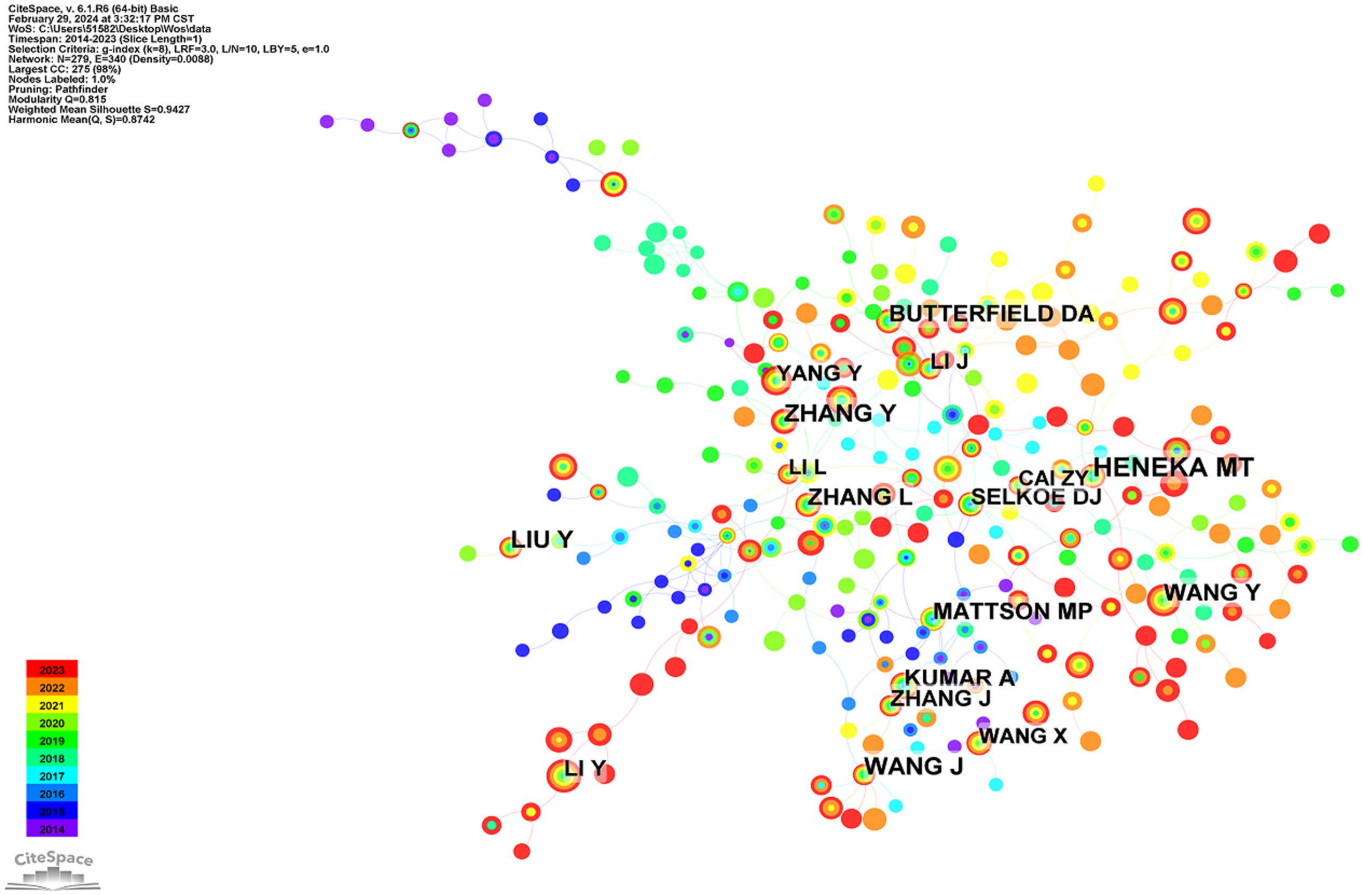
The network map of co-cited authors about NF-κB in CI.

Figure 8 shows the network map of co-cited authors, and Table 4 lists the top 10 co-cited authors with the highest frequency and centrality. Heneka received the most citations (*n* = 162), with the most citations occurring in 2021. Heneka’s research and co-cited papers focus on microglia, neuroinflammation, brain-derived neurotrophic factors, and Alzheimer’s disease (29–31). Selkoe achieved the highest centrality (0.93), suggesting that his research field had large-scale collaboration and cross-integration with other disciplines (32–34). More researchers should be encouraged to conduct high-quality studies in the future.

**Table 4:** The top 10 co-cited authors with the highest frequency and centrality.

Rank	Author	Citations	Author	Centrality
1	Heneka Mt	250	Selkoe Dj	0.93
2	Butterfield Da	160	Glass Ck	0.59
3	Mattson Mp	152	Heneka Mt	0.39
4	Wang J	151	Rohn Tt	0.33
5	Liu Y	129	Lee Yj	0.3
6	Wang Y	129	Awasthi A	0.29
7	Zhang Y	127	Perry Vh	0.27
8	Kumar A	125	Qin Ly	0.27
9	Selkoe Dj	120	Zhang H	0.25
10	Mcgeer Pl	110	Akiyama H	0.24

### 3.7 Analysis of co-occurrence, clustering, and citation burst of references

**Figure 9:**
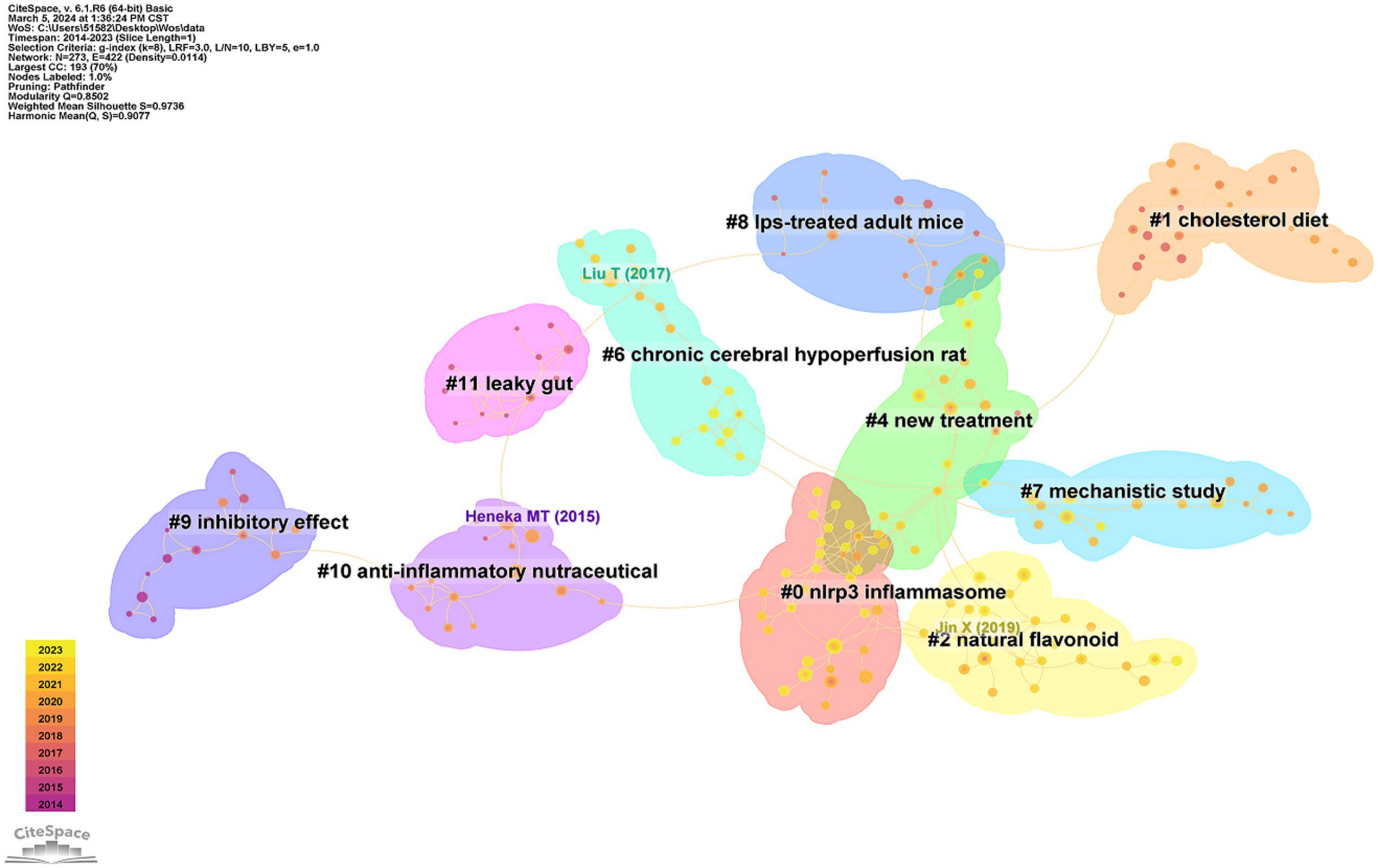
The main 10 clusters of co-reference about NF-κB in CI.

Co-citation analysis facilitates the identification of the knowledge base and influential articles within a large volume of references, enabling a more comprehensive exploration of the field’s development. A total of 10 clusters with modularity (Q) and weighted mean silhouette (S) values of 0.8502 and 0.9736, respectively, were identified in this study, indicating that the cluster results were convincing. Figure 9 visually displays the 10 clusters. The largest cluster (Cluster #0, labeled by the nlrp3 inflammasome) included 29 references, followed by Cluster #1 (labeled by cholesterol diet) with 26 references and Cluster #2 (labeled by natural flavonoid) with 25 references. Cluster #4 focussed on new treatments, such as dietary plant polyphenols, activating BDNF expression, and gut microbiota composition, and had 22 references. Cluster #6 (chronic cerebral hypoperfusion rat) had 18 references, Cluster #7 (mechanistic study) had 16 references, Cluster #8 (LPS-treated adult mice) had 15 references, Cluster #9 (inhibitory effect) had 15 references, and Cluster #10 (anti-inflammatory nutraceutical) had 14 references.

Table 5 shows the top 10 co-cited references with high frequency and high centrality and includes five studies and five reviews. Heneka published the most frequently cited articles, suggesting that neuroinflammation may drive the pathogenesis of Alzheimer’s disease and that microglial activation plays a key role (29). Liu et al. discussed the activation of NF-κB in association with inflammation and suggested that NF-κB induces the expression of several pro-inflammatory genes (35). Jin et al. explored a new treatment in which BAI was a promising neuroprotective compound for use in the prevention and treatment of microglia-mediated neuroinflammation during the progression of Alzheimer’s disease (36). Ising et al. concluded that the incidence of Alzheimer’s disease is due to microglia and NLRP3 inflammasome activation in the pathogenesis of tauopathies (31). Kinney et al. reported how to regulate microglia to improve the prospects of patients with AD (37).

**Table 5:** The top 10 co-cited references with high frequency and high centrality.

Rank	First author	Count	Centrality	Year	Cited reference	Journal	2022 JCR (IF)
1	Heneka MT	40	0.13	2015	Neuroinflammation in Alzheimer’s disease	Lancet Neurol	Q1 (12.82)
2	Liu T	37	0.03	2017	NF-κB signaling in inflammation	Signal Transduct Target Ther	Q1 (3.7)
3	Jin X	33	0.06	2019	Baicalin mitigates cognitive impairment and protects neurons from microglia-mediated neuroinflammation via suppressing NLRP3 inflammasomes and TLR4/NF-κB signaling pathway	CNS Neuroscience and Therapeutics	Q1 (5.5)
4	Ising C	23	0.14	2019	NLRP3 inflammasome activation drives tau pathology	Nature	Q1 (11.43)
5	Kinney Jefferson W	22	0.02	2018	Inflammation as a central mechanism in Alzheimer’s disease	Alzheimers and Dementia	Q1 (14)
6	Liddelow SA	21	0.02	2017	Neurotoxic reactive astrocytes are induced by activated microglia	Nature	Q1 (64.8)
7	Rehman SU	21	0.01	2017	Anthocyanins Reversed D-Galactose-Induced Oxidative Stress and Neuroinflammation Mediated Cognitive Impairment in Adult Rats	Molecular neurobiology	Q2 (5.1)
8	Tönnies E	20	0.21	2017	Oxidative Stress, Synaptic Dysfunction, and Alzheimer’s Disease	Journal of Alzheimer’s Disease	Q2 (3.5)
9	Zhao JY	19	0.05	2019	Neuroinflammation induced by lipopolysaccharide causes cognitive impairment in mice	Scientific Reports	Q2 (4.6)
10	DeTure MA	19	0	2019	The neuropathological diagnosis of Alzheimer’s disease	Molecular Neurodegeneration	Q1 (18.9)

Other frequently cited references included the study by Liddelow et al., who found that activated microglia induce neurotoxic reactive astrocytes, which are abundant in various human neurodegenerative diseases including Alzheimer’s disease, Parkinson’s disease, and multiple sclerosis (38). Rehman et al. also explored a novel treatment with anthocyanins that effectively attenuated neuroinflammation-mediated memory impairment and CI. The results of this previous study suggested that anthocyanins are a promising anti-neuroinflammatory agent for neurodegenerative diseases such as Alzheimer’s disease (39). Tönnies et al. emphasized the involvement of oxidative stress in the development of AD and the dual role of ROS in therapeutic strategies for neurodegenerative diseases (40). Zhao et al. revealed that inflammation is involved in the progression of CI by stimulating microglia through activation of the NF-κB signaling pathway (41). DeTure et al. provided an overview of Alzheimer’s disease pathology, which may affect the diagnosis and treatment.

Seven of these 10 previous studies were in the Q1 region (42). Basic research and mechanism research studies were mainly concentrated in microglia and Alzheimer’s disease. The subject with the highest reference centrality was oxidative stress, indicating that this field is closely integrated with other disciplines.

The concept of reference bursts suggests that scholarly attention should focus on specific articles within a particular timeframe in a given research field. Figure 9B displays the top 20 references with the strongest citation bursts. Generally, the most co-cited references had the strongest citation bursts, including studies by Zhao, Muhammad, Kinney, Duncombe, and DeTure, indicating the emerging trends or increasing interests in the field.

## 4 Discussion

### 4.1 Summary of basic information

In this study, a bibliometric analysis was conducted using VOSviewer and CiteSpace to identify the development trends and hotspots of research regarding the role of NF-κB in CI. A total of 1,468 original articles and reviews published from 2008 to 2023 were collected from the core collection of the Web of Science database. Publications regarding the role of NF-κB in CI began in 2010 and increased sharply in 2018. This increase may be due to the fact that the number of patients with dementia increased with the trend of global aging or due to the fact that treatments based on previous mechanism-based studies had little effect on dementia. Therefore, more researchers investigated the role of NF-κB in CI.

The core author analysis revealed that Hong Hao published the most studies in this field, with a total of 19 articles. The top three authors were observed in the largest cluster together (Figure 3A) and focussed on the mechanisms of NF-κB in CI. However, the most recent publication from these authors was in 2019. Five Chinese authors were included among the top 10 authors, which is consistent with the country analysis. Among the 77 countries represented in this study, China published the most literature, followed by the United States and South Korea. Therefore, China has a dominant position in the research field regarding the role of NF-κB in CI. The USA had the highest total link strength, indicating that the USA has strong influence and the most international cooperation. China Pharmaceutical University published the most studies in this field and was in a different cluster than other core institutions of Cairo University, Huazhong University Science and Technology, and China Medical University. The timing of the research conducted at each institution is represented in the figures. Famous institutions, such as Harvard University, have published studies regarding the role of NF-κB in CI, suggesting that this research is very valuable and promising (Figure 4C).

### 4.2 Research hotspots

Based on the visualization analysis of keywords, co-cited authors, and references, several research hotspots were identified.

#### 4.2.1 CI and Alzheimer’s disease

With the sharp increase in the elderly population, CI has become a research hotspot. The United Nations Department of Economic and Social Affairs Population Division predicts that the number of elderly people will reach 1.4 billion by 2030 (43). The concept of CI is broad and includes mild-to-severe CI, such as Alzheimer’s disease, vascular CI, mixed (Alzheimer’s and vascular), and dementia with Lewy bodies (44). Mild CI showed a strong citation burst in this study. Mild CI refers to CI that does not meet the diagnostic criteria for dementia. Until 1999, mild CI was considered a precursor to Alzheimer’s disease. In recent years, it has been determined that not all mild CI develops into Alzheimer’s disease (45). Alzheimer’s disease plays a pivotal role in the study of CI. The Web of Science Core Collection includes 139,122 studies regarding CI and 129,767 studies regarding Alzheimer’s disease. Therefore, Alzheimer’s disease is a hotspot in the CI research field.

#### 4.2.2 Mechanisms

##### 4.2.2.1 Oxidative stress

Sies first proposed the concept of oxidative stress in 1985 (46). It is widely used in the fields of biology and medicine (47). Oxidative stress may be a key mechanism leading to CI and neurodegenerative diseases (48). The brain is highly sensitive and susceptible to oxidation due to complex and interconnected pathologies, such as unsaturated lipid enrichment, glucose, mitochondria, calcium, and redox signaling that render the brain susceptible to oxidative stress (49). Previous studies have shown that the damage caused by oxidative stress, including damage to the immune system, is caused by the activation of NF-κB (50). These findings suggest that the pathogenesis of CI is multi-faceted.

##### 4.2.2.2 Inflammation and NF-κB

Inflammation is an important pathogenesis of CI (51), and the NF-κB signaling pathway is a classic inflammatory pathway *in vivo* (15). The NF-κB signaling pathway is mostly inactive. NF-κB protein is inhibited by IKB, and IKB kinase (IKK) phosphorylates the inhibitory protein IKB to ubiquitinate IKB and activate the NF-κB signaling pathway (52). In the classical NF-κB pathway, upstream inflammatory factors (53) induce IKK-mediated phosphorylation of IKB at ser32 and 36, activating downstream inflammatory genes and apoptosis suppressor genes (IAPs) (54). IAPs are major regulators of inflammatory responses (55). Inflammatory injury is a key link in the pathogenesis of cognitive dysfunction, which causes neuronal damage and activates the immune response (56).

##### 4.2.2.3 Microglia and Neuroinflammation

Microglia are the main effectors of inflammation in the central nervous system. As the first line of defense in the central nervous system, microglia play an important role in inflammatory responses and are regulated bidirectionally. Microglia perform the function of macrophages, including the phagocytosis of damaged cell debris and the removal of antigenic substances (12). In addition, microglia activate and release cytokines such as IL-1, promote the proliferation of astrocytes, and produce inflammatory factors such as NO and TNF-*α* that cause nerve damage (57). Neuroinflammation is closely related to nerve damage and disrupts neural function, ultimately resulting in CI (58). Activated microglia are a pathological feature of neuroinflammation observed in the setting of CI (36).

### 4.3 Future research trends

“Neuroinflammation,” “microglial,” and “pathway” remain hotspots of research regarding the role of NF-κB in CI (59). Research regarding CI is more inclined toward vascular dementia caused by stroke, which may be due to the reversible nature of vascular dementia as early interventions can achieve good social and economic results (60). “Therapeutic target” is a hotspot keyword from 2021, and the gut microbiome is a therapeutic target for CI. The gut–brain axis is closely associated with cognition (61). Lipopolysaccharides are another hotspot for future research and are thought to act as a mediator between the gut microbiome and CI (62) and to induce neuroinflammation leading to CI (63). In conclusion, the future trend of research regarding the role of NF-κB in CI is integrated and systematic. New elements are constantly added to classical research, such as apolipoproteins and tau proteins, which are mostly included in research regarding neurodegenerative diseases (64). At the same time, more public funding is needed for basic research of NF-κB in CI. Multidisciplinary collaboration and more publications on cognitive impairment are encouraged to guide clinics and enable large-scale clinical research through basic research findings.

### 4.4 Study limitations

This study is not without limitations. First, the most recent studies regarding the role of NF-κB in CI were excluded, which may lead to a hysteretic quality. Second, only the core collection of the Web of Science was included in the literature search; therefore, important literature from other databases may have been overlooked. Third, the CiteSpace data analysis process parameters must be defined separately, which affects the accuracy of the data analysis to a certain extent. Fourth, due to the limitations of the VOSviewer and CiteSpace software, the data analysis is not sufficiently comprehensive. Future studies should include non-English literature and literature from other sources.

## 5 Conclusion

This is the first bibliometric analysis regarding the role of NF-κB in CI in which VOSviewer and CiteSpace were used. The results of this bibliometric study indicate that research regarding NF-κB in CI is currently growing at a rapid pace. China has published the most papers in this field, and its research potential in this area is huge. The United States remains the biggest influence in this area. The role of oxidative stress, inflammation, and microglia in NF-κB signaling was a hotspot of CI research. Emerging studies have focused on therapeutic targets, microbiota, and ketogenic diets. Collaborative research is necessary in this field.
